# Assessment of *Torquetenominivirus* (TTMV) and *Torquetenomidivirus* (TTMDV) as Complementary Biomarkers to *Torquetenovirus* (TTV)

**DOI:** 10.3390/ijms26031022

**Published:** 2025-01-25

**Authors:** Lilia Cinti, Pietro Giorgio Spezia, Piergiorgio Roberto, Gianluca Russo, Quirino Lai, Carolina Carillo, Federica Frasca, Guido Antonelli, Fabrizio Maggi

**Affiliations:** 1 Laboratory of Microbiology and Virology, Department of Molecular Medicine, Sapienza University of Rome, Viale Regina Elena, 324, 00161 Roma, Italy; lilia.cinti@uniroma1.it (L.C.); federica.frasca@uniroma1.it (F.F.); guido.antonelli@uniroma1.it (G.A.); 2 PhD National Interest in Innovation in the Diagnosis, Prevention and Treatment of Infections at Epidemic-Pandemic Risk, Department of Medical Biotechnologies, University of Siena, 53100 Siena, Italy; 3 University Hospital “Policlinico Umberto I”, 00161 Rome, Italy; gianluca.russo@uniroma1.it (G.R.); quirino.lai@uniroma1.it (Q.L.); carolina.carillo@uniroma1.it (C.C.); 4 Laboratory of Virology, National Institute for Infectious Diseases Lazzaro Spallanzani-IRCCS, 00149 Rome, Italy; pietro.spezia@inmi.it (P.G.S.); fabrizio.maggi@inmi.it (F.M.); 5 PhD National Programme in One Health Approaches to Infectious Diseases and Life Science Research, Department of Public Health, Experimental and Forensic Medicine, University of Pavia, 27100 Pavia, Italy; 6 Department of Public Health and Infectious Diseases, Sapienza University of Rome, 00161 Rome, Italy; 7 Department of General and Specialistic Surgery, Sapienza University of Rome, 00161 Rome, Italy; 8 Department of General and Specialistic Surgery “Paride Stefanini”, Sapienza University of Rome, 00161 Rome, Italy

**Keywords:** *Torquetenovirus*, *Torquetenominivirus*, *Torquetenomidivirus*, anellovirus, COVID-19, SOT, biomarker

## Abstract

Recent studies have identified *Torquetenovirus* (TTV) as a promising biomarker of immune competence, particularly in assessing the vaccine response of solid organ transplant (SOT) recipients. However, given the individual variability of viral load, it is not yet possible to define "normal levels”. Nevertheless, TTV is just one component of the broader *Anelloviridae* family, which also includes *Torquetenominivirus* (TTMV) and *Torquetenomidivirus* (TTMDV). This study explores whether the viremia of TTMV and TTMDV offers a stronger predictive marker for vaccine efficacy in SOT recipients. A cohort of 168 SOT patients (142 kidney and 26 lung transplant recipients) who received the BNT162B2 mRNA vaccine was examined, with viral loads quantified through virus-specific real-time PCR. While TTV remains a potentially useful biomarker for evaluating immune response, the combined analysis of all anelloviruses viremia provides deeper insights, particularly in cases where TTV is undetectable. Notably, only TTMV exhibited a pattern similar to TTV, suggesting its potential as an alternative biomarker when TTV is absent from the patient’s virome.

## 1. Introduction

The COVID-19 pandemic has posed unprecedented challenges to global healthcare, particularly for solid organ transplant (SOT) recipients who are immunocompromised and exhibit diminished vaccine responses [1,2,3]. Identifying reliable biomarkers to predict vaccine efficacy in this vulnerable population is critical.

*Torquetenovirus* (TTV), a small single-stranded DNA virus belonging to the *Anelloviridae* family, is widely prevalent and considered part of the human virome [4,5]. Its replication is regulated by a functioning immune system, and its plasma viral load is thought to reflect the degree of immunosuppression [6,7,8]. High TTV loads have been strongly associated with an increased risk of infection, while low TTV loads have been linked to an elevated risk of organ rejection in transplant patients, making it a valuable marker for monitoring immune status in this population [9,10,11,12]. In addition to TTV, the *Anelloviridae* family includes other related viruses, such as *Torquetenominivirus* (TTMV) and *Torquetenomidivirus* (TTMDV). Although these viruses share numerous genomic characteristics with TTV, their genome length is a key feature that differentiates them [13,14]. Anelloviruses are highly prevalent in the human population, with over 80% of individuals carrying viral DNA in their plasma. While largely considered non-pathogenic, their widespread presence underscores their potential relevance to immune function. However, given the individual variability of viral load, it is not yet possible to define "normal levels”. Recently, TTV has emerged as a promising marker of immune competence and vaccine responsiveness in SOT patients [15,16,17,18]. However, the broader role of total anellovirus viremia (TAV)—encompassing TTV, TTMV, and TTMDV—as a more comprehensive predictor of immune response remains unexplored. This study investigates whether TAV offers a superior measure of SARS-CoV-2 vaccine effectiveness compared to TTV viremia alone in SOT recipients, with the aim of refining vaccination strategies and improving patient outcomes.

## 2. Results

The study involved 168 patients who had received SOT. Serum samples from each patient were collected, on average, 55 days following the administration of the second dose of mRNA-based vaccine BNT162B2 against SARS-CoV-2. Among the participants, 142 had received a kidney transplant and the remaining 26 had received a lung transplant. The cohort comprised 58% males and 42% females, with a median age of 56 years (IQR = 48–65 years). Vaccination elicited a significant antibody response in 88 out of 168 patients, with a mean antibody level of 1017 BAU/mL (IQR = 93–901 BAU/mL).

To examine a possible link with the immune response triggered by SARS-CoV-2 vaccination, DNA of TTV, TTMV, and TTMDV was detected and measured in all patients. The viral loads were then analyzed to compare individuals who responded to the vaccine and those who did not.

**Figure 1:**
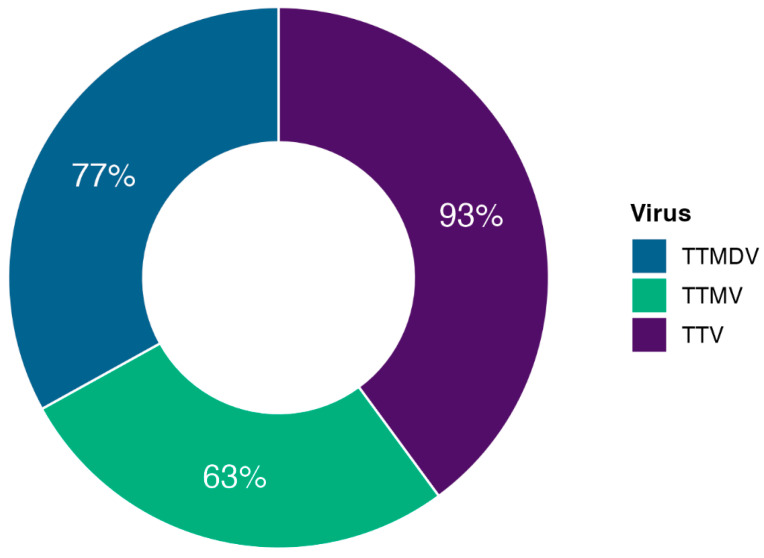
Prevalence rates of TTV, TTMV, and TTMDV in the study population.

**Figure 2:**
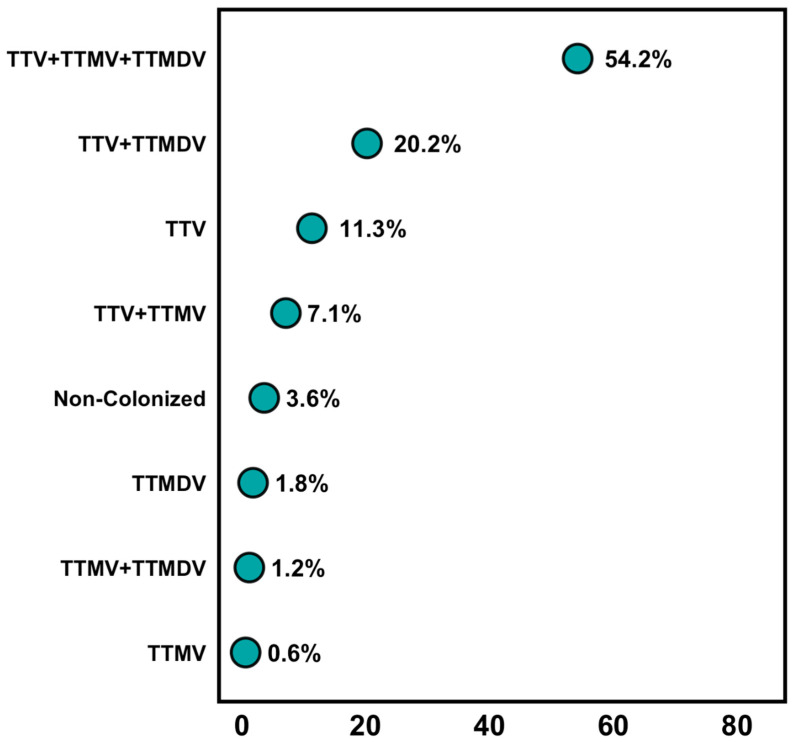
Co-infection events in the studied population: the y-axis lists the co-infection events, while the x-axis indicates their abundance percentages. (Abbreviations: TTV = *Torquetenovirus*; TTMV = *Torquetenominivirus*; TTMDV = *Torquetenomidivirus*. The “+” sign indicates co-infection between the respective viruses).

As shown in Figure 1, TTV had the highest prevalence among the study participants, with a detection rate of 93%. This was followed by TTMDV, which was found in 77% of the patients, and TTMV was detected in 63%. The study was carried out at the individual patient level to identify the presence of any of the three viruses (Figure 2). The results showed that 54.2% of the patients had all three viruses detected. Patients with only TTMV and TTMDV comprised 1.2%, those with solely TTV and TTMV accounted for 7.1%, and the co-occurrence of TTV and TTMDV was found in 20.2%. The exclusive detection of TTV, TTMDV, or TTMV occurred in 11.3%, 1.8%, and 0.6% of the patients, respectively. Furthermore, 3.6% of the patients showed no presence of any of the three target viruses. Consequently, considering the presence of at least one of the three anelloviruses, the coverage of the tested population reaches up to 96.4%.

**Figure 3:**
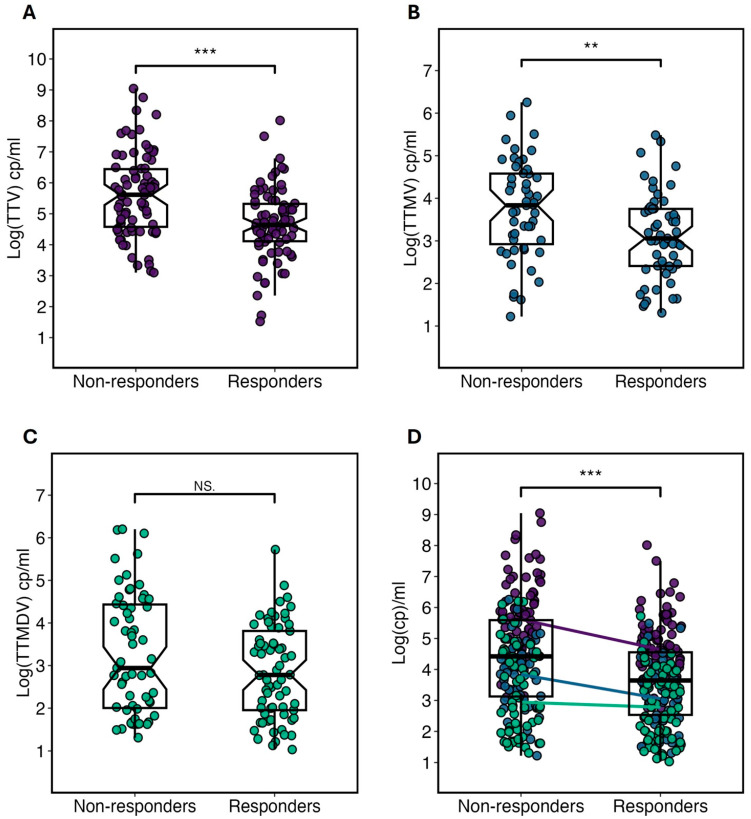
The chart illustrates the viral loads for (**A**) TTV, (**B**) TTMV, (**C**) TTMDV, and (**D**) an overlay of TTV, TTMV, and TTMDV in patients who either responded or did not respond to SARS-CoV-2 vaccination. Medians of the respective viral loads are displayed in all box plots. Statistical significance is indicated as follows: *p* > 0.05 (NS), *p* < 0.01 (**), *p* < 0.001 (***). The exact *p*-values are as follows: (**A**) *p* = [5 × 10^−5^], (**B**) *p* = [4.7 × 10^−4^], (**C**): *p* = [0.12], (**D**): *p* = [7 × 10^−6^].

The viral loads of different anelloviruses were assessed against patients’ antibody responses to vaccination (Figure 3). A higher median TTV copy number was found in non-responders (5.6 log copies/mL) compared to responders (4.7 log copies/mL), showing strong statistical significance (*p* = 5 × 10^−5^) (Figure 3A). Similarly, TTMV viremia displayed higher values in non-responders than responders (median 3.8 log vs. 3.0 log copies/mL, *p* = 4.7 × 10^−4^) (Figure 3B). No significant difference was seen for TTMDV between the groups (median 3.1 log vs. 2.8 log copies/mL, *p* = 0.12) (Figure 3C). Finally, the 3D graph presents an overlay of the viral loads of the three anelloviruses, with each patient plotted multiple times based on the co-infections they harbor (defined as the simultaneous colonization of more than one anellovirus species in the same patient). By simultaneously analyzing the viral loads of multiple viruses for each individual, we achieved a more robust statistical significance (*p* = 7 × 10^−6^) compared to all other single-virus analyses. Additionally, this visualization allows for a clearer comparison of viral load levels: TTV reaches the highest viral loads, followed by TTMV, and lastly, TTMDV.

**Figure 4:**
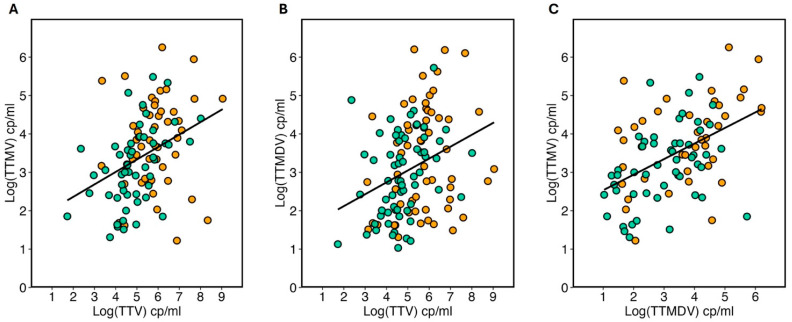
The diagram highlights linear correlations among: (**A**) TTMV and TTV, (**B**) TTMDV and TTV, (**C**) TTMV and TTMDV. Non-responsive patients to the SARS-CoV-2 vaccine are marked in orange, while seroconverted patients appear in green. The black regression line indicates the correlation level between the viral loads analyzed.

The study also examined the correlation between the copy numbers of various viruses. As depicted in Figure 4, there was a statistically significant positive correlation (*p* < 0.0001) across all virus pairs. The Spearman coefficients (r) were 0.42, 0.39, and 0.47 for TTV vs. TTMV, TTV vs. TTMDV, and TTMV vs. TTMDV, respectively, indicating a weak to moderate correlation.

**Figure 5:**
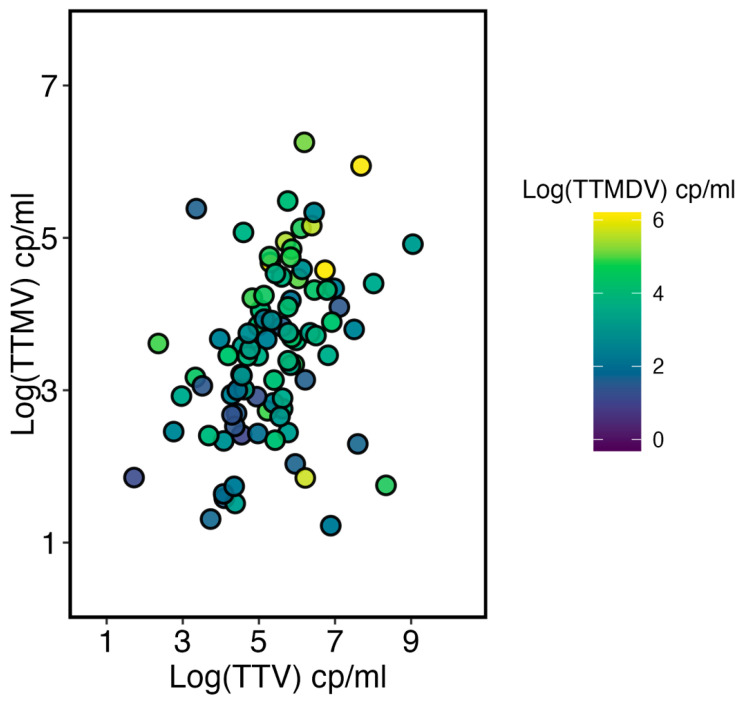
The scatter plot displays the relationship between the viral loads of the three viruses studied, measured in Log copies/mL. Only patients possessing all three viral species are shown. The color scale, as indicated in the legend, represents the TTMDV viral load.

Finally, the viral load correlation among all studied viruses was examined. Figure 5 shows that as TTV and TTMV loads rise, there is a slight increase in the TTMDV load. Despite this, the TTMDV load does not seem strongly correlated with TTV or TTMV concentrations.

## 3. Discussion

There is a growing interest in investigating the role of TTV as an indicator of immune status in immunocompromised patients, with particularly promising results observed in studies involving patients undergoing SOT [19,20,21,22]. In our previous research [15], we found intriguing correlations between TTV load and seroconversion following SARS-CoV-2 vaccination in a cohort of SOT patients. In this study, we aimed to examine the presence and loads of two other anelloviruses, TTMV and TTMDV, within the same patient group to gain a deeper understanding of the relationship between these anelloviruses, which constitute a significant component of the human virome [23], and the immune response of the infected host [24,25].

Firstly, a direct link was found among the viral loads of various anelloviruses, indicating that an increase in one virus’ viral load is likely followed by a rise in others. These initial data suggest that the *Anelloviridae* family experiences similar immunological regulation within the host.

After proving that TTV can distinguish between responders and non-responders to SARS-CoV-2 vaccination, we investigated if TTMV and TTMDV show similar behavior. TTV remains the best candidate, with TTMV showing a comparable trend but being less frequent in the group studied, resulting in slightly lower statistical significance. TTMDV differs from both viruses; although it is more frequently detected than TTMV, its viral load cannot be used to differentiate responsiveness to the vaccination. This discrepancy could be due to weak correlation data, suggesting other interpretations for the coexistence of these conditions.

In addition to evaluating the prevalence of the three anelloviruses within our population, we investigated the occurrence of potential co-infections in individuals. The findings were quite fascinating. Most of the population is co-infected with all three viruses. Co-infections involving two viruses are significantly more common when TTMV or TTMDV is paired with TTV, compared to instances without it. This pattern is also observed in single-virus infections, where the presence of TTMV or TTMDV alone is exceedingly uncommon.

The high prevalence of TTV, as frequently reported in the literature [26,27], could indicate the virus’s enhanced ability to persist in the host, higher transmissibility, or simply more effective detection compared to other family members. Another noteworthy point arises with the 7.2% of patients who showed no TTV infection. When research is extended to include the other two viruses, the overall prevalence of anelloviruses in the total population increases to 96.4%. Although this represents a modest rise, it clearly shows that assessing TTMV and TTMDV can be valuable when TTV is undetected. Indeed, a study by Gorzer et al. [28] indicated that PCR techniques may overestimate TTV levels due to the amplification of sequences from other anelloviruses. Notably, all PCR methods tested so far show similar effectiveness in linking TTV load with transplant rejection and infection risks. This suggests that despite potential cross-reactions, the clinical utility of these tests remains unaffected. This would support the hypothesis that evaluating the viral loads of other anelloviruses could provide a more comprehensive assessment of immune system function. However, it is important to note that the test remains meaningful when a subject is colonized by multiple anelloviruses or by TTV or TTMV alone. In the rare cases where only the TTMDV genome is amplified, the test’s significance would be weakened.

In conclusion, our study suggests the potential of a comprehensive evaluation of the *Anelloviridae* family, particularly through the analysis of TTV, TTMV, and TTMDV viral loads, as a valuable tool for immune monitoring in immunocompromised patients. While TTV remains a potentially useful biomarker for assessing immune response, the inclusion of TTMV and TTMDV in the evaluation framework could offer a more nuanced understanding, especially in cases where TTV is undetectable. However, in our study, only TTMV demonstrated a pattern akin to TTV, indicating its potential as a viable surrogate when TTV is absent from the patient’s virome. These findings suggest the value of broadening viral monitoring to include additional anelloviruses, offering a more comprehensive assessment of immune competence. However, our results show that TTV/anellovirus levels can indicate population norms for immunocompetence, but the wide range of these values makes it difficult to set a cutoff for predicting individual vaccine response.

Further research is necessary to better understand the role of anelloviruses load in evaluating immune status, such as by comparing an individual’s values of anelloviruses load before and after the initiation of immunosuppression. Additionally, a more in-depth assessment is required to determine the potential of TTMDV to distinguish other immunological profiles.

## 4. Materials and Methods

### 4.1. Patients and Specimens

A retrospective study was conducted on 168 SOT patients, comprising 142 kidney and 26 lung transplant recipients who were followed as outpatients at Policlinico Umberto I Hospital of Rome, at the Department of General and Specialistic Surgery. Serum samples were collected, on average, 55 days following the administration of the second dose of the mRNA-based vaccine BNT162B2 [29]. For consistency, the study only included samples from patients who did not have a declared or documented SARS-CoV-2 infection. Additionally, patients who never showed a detectable TTV load during the study were excluded from the statistical analysis, as one negative sample does not exclude the possibility of infection. The study was conducted according to the guidelines of the Declaration of Helsinki (https://www.wma.net/news-post/revised-declaration-of-helsinki-adopted-by-the-global-medical-community-strengthening-ethical-standards-in-clinical-research-involving-humans/, accessed on 23 January 2025) and approved by the Local Ethics Board of the Sapienza University of Rome (CE 6338). Informed consent was obtained from each participating patient.

### 4.2. Anellovirus DNA Detection and Quantification

The total DNA was isolated from 300 μL of serum samples processed with the NucliSens easyMAG extractor (BioMérieux) according to the manufacturer’s instructions. The genomes of TTV, TTMV, and TTMDV were identified using the CFX96 platform from Bio-Rad Laboratories, Inc., Hercules, CA, USA. Real-time PCR assays were optimized for the amplification of the TTV, TTMV, and TTMDV genomes using the Takara Premix Ex Taq kit (Takara Bio Inc., Kusatsu, Shiga, Japan). The specific primer pairs and probe sequences and their positions on the reference genome for each virus are listed in Table 1. The primers for TTV, TTMV, and TTMDV were used at concentrations of 0.9 μM, 0.9 μM, and 0.7 μM, respectively, while the probe was used at a concentration of 0.1 μM for all reactions. The primers for TTV were designed based on previously published sequences [30]. The TTMV and TTMDV reactions were optimized and tested for specificity by Sanger sequencing of the obtained fragments. The sequences obtained were aligned and compared with known sequences in the GenBank database to ensure the correct identification of the viral genomes. Quantitative assays for TTV, TTMV, and TTMDV were developed using specific standards based on reconstructed plasmids (pJET 2.1 plasmid, Thermo Fisher, Segrate (MI), Italy) that included the cloned target amplification region for each virus’s real-time PCR reaction.

### 4.3. Antibody Response to SARS-CoV-2

The antibody response to the BNT162B2 vaccine was evaluated using the chemiluminescence technology of the LIAISON^®^ SARS-CoV-2 TrimericS IgG immunoassay developed by DiaSorin S.p.A. (Saluggia, Italy). The IgG titers were expressed in binding antibody units/mL (BAU/mL). The assay’s detection limit for antibody titer in serum is 4.81 BAU/mL. According to the manufacturer’s instructions, values falling within the range of 4.81–33.8 BAU/mL were interpreted as negative, while values >33.8 BAU/mL were considered positive.

### 4.4. Statistical Analysis

Data management, analysis, and visualization were performed using R version 4.3.x and RStudio version 2024.09.1-478 (https://www.rstudio.com/). Null hypothesis testing was performed using the non-parametric Mann–Whitney–Wilcoxon test while to assess the correlation between the data, the non-parametric Spearman’s rank correlation test was employed. The strength of the correlation was interpreted using standard intervals for the Spearman correlation coefficient as follows: <0.40 weak correlation, 0.40–0.59 moderate correlation, and >0.59 strong correlation. Correlation values were considered significant if the *p*-value associated with the Spearman test was below the significance level of 0.05. *p*-values are reported in figures according to the following notation: *p* < 0.05 (*), *p* < 0.01 (**), and *p* < 0.001 (***).

## Figures and Tables

**Table 1:** Primer and probe 5′- > 3′ nucleotide sequences, reference nucleotide positions, and PCR thermal profile conditions for the detection of TTV, TTMDV, and TTMV. The same thermal cycling conditions were used for all three viruses, and FAM was used for probe detection.

**TTV**		
		**Ref. nt position (NC_002076.2)**
Forward primer	GTG CCG IAG GTG AGT TTA	177–194
Reverse primer	AGC CCG GCC AGT CC	226–239
TaqMan Probe	FAM/TCA AGG GGC AAT TCG GGC T/BHQ1	205–223
Thermal profile condition	95 °C 1 min, 45 cycles of 95 °C for 5 s and 50 °C for 30 s (FAM detection)	
**TTMDV**		
		**Ref. nt position (NC_009225.1)**
Forward primer	TGC AGG GAC CGG ATC GAG C	68–86
Reverse primer	AAT TGC CCC TAG ACC TCG GT	141–160
TaqMan Probe	FAM/ACT CAC CTC GGG CTC CCG CCC AT/BHQ1	112–134
Thermal profile condition	95 °C 1 min, 45 cycles of 95 °C for 5 s and 58 °C for 30 s (FAM detection)	
**TTMV**		
		**Ref. nt position (NC_025726.1)**
Forward primer	GGA TCA CTT CAG TGA CTC CAG	209–229
Reverse primer	AGT TTC TTG CCC GTT CCG CCA	301–321
TaqMan Probe	FAM/AGG TGA GTG AAA CCA CCG TAG TCT AG/BHQ1	252–277
Thermal profile condition	95 °C 1 min, 45 cycles of 95 °C for 5 s and 60 °C for 30 s (FAM detection)	

FAM = 6-carboxyfluorescein; BHQ1 = Black Hole Quencher 1; I = Inosine.

## Data Availability

The data cannot be publicly shared as they contain sensitive information that could compromise the privacy and consent of research participants. However, interested parties can request access to the data from the corresponding author. This restriction is in place to uphold privacy and ethical standards.
